# The association between diabetes mellitus, glucose, and chronic musculoskeletal complaints. Results from the Nord-Trøndelag Health Study

**DOI:** 10.1186/1471-2474-9-160

**Published:** 2008-12-02

**Authors:** Ole M Hoff, Kristian Midthjell, John-Anker Zwart, Knut Hagen

**Affiliations:** 1 Department of Neuroscience, Faculty of Medicine, Norwegian University of Science and Technology, Trondheim, Norway; 2 HUNT research centre, Department of Public Health, Faculty of Medicine, Norwegian University of Science and Technology, Trondheim, Norway; 3 Norwegian National Headache Centre, Section of Neurology, St. Olav's Hospital, Trondheim, Norway; 4 Department of Neurology, Ullevål University Hospital, and Faculty of Medicine, University of Oslo, Oslo, Norway

## Abstract

**Background:**

The relationship between diabetes mellitus (DM) and chronic musculoskeletal complaints (MSCs) is unclear. The aim of this study was to investigate the association between DM, non-fasting glucose and chronic MSCs defined as pain and/or stiffness ≥ 3 months during the past year in the general adult population.

**Methods:**

The results were based on cross-sectional data from 64,785 men and women (aged ≥ 20 years) who participated in the Nord-Trøndelag Health Survey, which included 1,940 individuals with known DM. Associations were assessed using multiple logistic regression, estimating prevalence odds ratio (OR) with 95% confidence intervals (CIs).

**Results:**

High non-fasting glucose was associated with a lower prevalence of chronic MSCs compared to a low glucose level. DM was associated with higher prevalence of chronic MSCs, in particular chronic widespread MSCs. In the multivariate analysis, adjusting for glucose level, BMI, age, gender and physical activity, chronic widespread MSCs was 1.6 times more likely (OR = 1.6, 95% CI 1.2–2.2) among individuals < 60 years of age with DM than among those without DM. The association between chronic widespread MSCs and DM was most evident among the group of individuals aged < 60 years with either type 2 DM or unclassified DM (OR = 1.8, 95% CI 1.3–2.5).

**Conclusion:**

In this cross-sectional study a high non-fasting glucose was associated with lower prevalence of chronic MSCs. Among individuals with known DM chronic widespread MSCs were more likely.

## Background

Musculoskeletal complaints (MSCs) are among the major health problems worldwide and the most frequent cause of long-term sickness leave in Norway [1,2]. Increased mortality has been reported among individuals with chronic widespread MSCs [3], which further emphasizes that this group of patients may constitute an important public health problem.

In Nord-Trøndelag County in Norway 45% of the adult population reported chronic MSCs during the last year [4]. The prevalence of known diabetes mellitus (DM) was 3% [5]. Despite that both disorders are relatively common, few studies have focused on the relationship between chronic MSCs and diabetes mellitus (DM). DM has been associated with increased fracture risk [6] and rotator cuff tendinitis [7]. Some studies have reported reduced pain thresholds in individuals with hyperglycemia [8,9], but the relationship between DM and chronic MSCs remains unclear.

To clarify the potential association between DM and chronic MSCs a cross-sectional study is a time effective first-step which also may provide clues to the pathophysiology of chronic MSCs. The main purpose of this large population-based cross-sectional study was to evaluate the association between DM, non-fasting glucose and chronic MSCs.

## Methods

Between 1995 and 1997, all inhabitants aged 20 years and above were invited to participate in the Nord-Trøndelag Health Survey (the HUNT study). Details of this comprehensive health study are described elsewhere [10]. In short, two questionnaires including more than 200 health-related questions were administered to the participants. The first questionnaire (Q1) was sent along with the invitation, and delivered when attending the health examination during which non-fasting blood samples were drawn. All participants received a second questionnaire (Q2) which was returned by postal mail. Out of the 92,936 invited individuals, 65,081 (70.0%) answered the first question about DM, whereas 64,785 (69.7%) responded to the first question about MSCs.

### Musculoskeletal complaints

Both Q1 and Q2 included questions about musculoskeletal symptoms adopted from the Standardized Nordic Questionnaire, which has previously been evaluated and found to give reliable estimates for low back and upper limb and neck discomfort, in particular for symptoms during the past year [11-13]. In Q1 participants were asked whether they had suffered pain or stiffness in muscles and joints lasting for at least 3 months during the last year, whereas in Q2, they were asked to indicate the number of days during the last month of such complaints. In both questionnaires, those participants who responded "yes" were then asked to tick off one or several of the following nine areas of the body; neck, shoulders, elbows, wrist/hands, upper back, low back, hips, knees, and/or ankles/feet.

In the present study, individuals with chronic MSCs (pain and/or stiffness ≥ 3 months during the past year) were subdivided into chronic widespread MSCs and chronic non-widespread MSCs. "Chronic widespread MSCs" were defined as pain and/or stiffness ≥ 3 month during the past year and ≥ 15 days with symptoms during the last month from all of the following regions: axial skeletal pain (pain in the neck, chest/abdomen, upper back, or lower back), pain above the waist (neck, shoulders, elbows, wrist/hands, chest/abdomen, or upper back) and below the waist (lower back, hips, knees, or ankles/feet). Individuals with chronic MSCs not fulfilling the criteria for chronic widespread MSCs were defined as having chronic non-widespread MSCs (mutually exclusive).

### Diabetes mellitus

Non-fasting glucose was drawn from more than 99% of the participants who attended the clinical examination. Fasting glucose, glycosylated hemoglobin (HbA1c), C-peptide and anti-glutamic acid decarboxylase (anti-GAD) was drawn from 74% of the participants with a positive answer to the question "Do you have or have you ever had diabetes?" in Q1 (known DM). Known DM was reported in a total of 1,940 (3%) out of the 64,785 participants who responded to the first question about MSCs. A total of 216 persons with no previous known DM had non-fasting glucose ≥ 11.1. Although a considerable proportion of these probably had previously undiscovered DM, these were included as a separate group in our analyses.

Patients starting insulin treatment within 6 months of diagnosis were classified as having type 1 DM (classic type 1), if, in addition, they were anti-GAD positive (≥ 0.08) or had C-peptide levels < 150 pmol/l (n = 122). Type 2 diabetic subjects were anti-GAD negative (< 0.08) and had not received insulin treatment within one year of diagnosis (n = 1120). LADA was defined by anti-GAD positivity and no insulin treatment within 12 months after diagnosis (n = 127). For gestational diabetes mellitus (GMD) the criteria were anti-GAD negative and no insulin treatment, and/or other information about diabetes during pregnancy (n = 12). The criteria for MODY were anti-GAD negative and debut < 25 years and diabetes in relatives and no insulin treatment started within 12 months after debut (n = 8). Only 20 persons fulfilled the criteria for MODY or GDM, and these were therefore merged together with the unclassified group, consisting of patients with incomplete information on insulin treatment and results of C-peptide and anti-GAD.

### Statistical analysis

Differences between continuous variables were tested with analyses of variance (one-way ANOVA) and between proportions by chi-squared test. In the multivariate analyses, using logistic regression, we estimated the prevalence odds ratio (OR) with 95% confidence interval (CI) for the association between chronic MSCs and DM. We adjusted for age, body mass index (BMI), gender and physical activity as confounders. Other potential confounding factors like education, occupation, smoking, previous myocardial infarction, alcohol consumption, and mean systolic blood pressure were also evaluated, but were excluded from the final analyses because they changed the OR by less than 5%. Serum glucose, HbA_1c_, and duration of DM were categorized into quartiles based on individual values, but in separate analyses also treated as a continuous variable. When appropriate, serum glucose was also treated as a single ordinal variable (categories 1 to 4 based on quartiles) and was incorporated in a two sided test for trend to evaluate the probability of a linear relationship between glucose categories and prevalence of MSCs. We also investigated potential interaction between age and diabetes status by including the product of the two variables into the logistic regression analyses. The interaction coefficient was tested using Wald χ^2 ^statistics. Data analyses were performed with the Statistical Package for the Social Sciences, version 15.0 (SPSS, Chicago, Illinois, USA)

The study was approved by the Regional Committee for Ethics in Medical Research, and the HUNT Study is also approved by the Norwegian Data Inspectorate.

## Results

Among the 64,785 participants, 30,157 (46.5%) reported chronic MSCs, whereof 3,240 (5.0%) had chronic widespread MSCs, and the remaining 26,917 (41.5%) chronic non-widespread MSCs. The prevalence of chronic MSCs increased with age with a peak in the age group 60–64 years (59.7%), and were higher for women than men in all age groups (overall 50.1% versus 42.6%, p < 0.001). The prevalence of chronic MSCs also increased with BMI with a peak among obese with BMI ≥ 30 kg/m^2 ^(54.6%), and were higher among the physically inactive than active individuals (55.4% versus 44.9, p < 0.001).

Educational level, level of physical activity, and proportion of smokers were lower among individuals with DM compared to those without DM, whereas, age, BMI, mean systolic BP, and prevalence of alcohol abstainers and myocardial infarction were significantly higher (p < 0.001) Table 1). The proportion of women did not differ significantly between the groups with or without DM (p = 0.20). Among individuals with DM, those with type 1 DM was youngest and most likely to be men (Table 1).

**Table 1:** Background data on persons without and with known diabetes mellitus (DM) (type 1, type 2, type 2, LADA, and other types/unclassified)

Variables	No DM	All DM types	Classical Type 1 DM	Type 2 DM	LADA	Other types/unclassified DM	Not DM, but glucose ≥ 11.1
n	62,626	1940	122	1120	127	571	216
Gender, female (%)	53.2	51.7	41.0	50.5	46.5	57.4	37.5
Mean age	48.4	64.4	47.5	67.1	67.4	63.1	61.6
Years of education ≥ 13 (%)	19.3	9.1	19.7	6.5	11.0	10.3	12.0
Mean BMI	26.3	29.0	26.1	29.6	28.5	28.6	29.5
Current smoking (%)	28.9	17.5	24.6	15.4	9.4	21.9	26.9
High physical activity* (%)	16.1	7.8	13.9	7.2	11.0	6.8	4.6
Alcohol abstainers (%)	12.2	30.8	13.7	32.0	36.1	31.0	17.2
Previous myocardial infarction	3.0	12.7	5.7	13.5	12.0	12.9	5.6
Mean systolic blood pressure	137	154	140	156	154	153	156

* High physical activity level = ≥ 3 hours/week with hard physical activity

**Figure 1:**
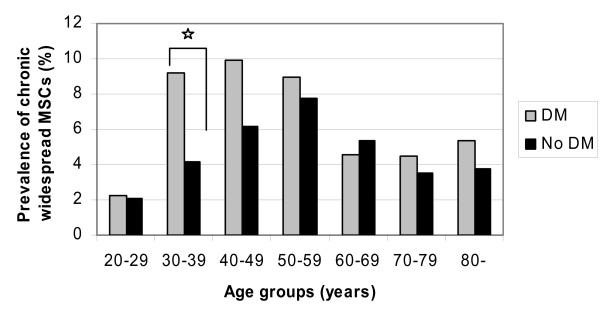
Prevalence (%) of chronic widespread MSCs by age group. * p < 0.05.

In the multivariate analyses, adjusting for age, BMI, gender and level of physical activity as confounding factors, prevalence of chronic MSCs was higher among patients with DM than among individuals without (OR = 1.2, 95% CI 1.1–1.3, Table 2). This association was most evident among individuals with chronic widespread MSCs (OR = 1.3, 95% CI 1.1–1.6). The difference in unadjusted prevalence of chronic widespread MSCs between individuals with or without DM was most prominent in those below 60 years of age (Fig. 1). Although no significant interaction was found between age and diabetes status in the multivariate analyses (p = 0.16), chronic widespread MSCs was 1.5 times more likely (OR = 1.5, 95% CI 1.1–2.1) among subjects with DM than among those without in a separate analysis for individuals < 60 years. Among those 60 years and older, the corresponding OR was 1.1 (0.8–1.5).

**Table 2:** Prevalence OR^# ^of musculoskeletal complaints (MSCs) related to DM and non-fasting glucose levels

Variables	Total	Chronic MSCs		Chronic widespread MSCs MSCs		Chronic non-widespread MSCs	
	64,785	%	OR (CI)	%	OR (CI)	%	OR (CI)
**DM**							
No	62,629	46.2	1.0 (reference)	5.0	1.0 (reference)	41.3	1.0 (reference)
Yes	1,940	56.9	1.2 (1.1–1.3)	5.9	1.3 (1.1–1.6)	51.0	1.1 (1.0–1.3)
No, but glucose ≥ 11.1	216	45.4	0.7 (0.5–0.9)	5.1	0.8 (0.4–1.5)	40.3	0.7 (0.5–0.9)
							
**DM**							
No	62,629	42.2	1.0 (reference)	5.0	1.0 (reference)	41.3	1.0 (reference)
Type 1	122	45.9	1.0 (0.7–1.5)	3.3	0.7 (0.2–1.9)	42.6	1.1 (0.7–1.6)
Type 2	1,120	57.7	1.1 (1.0–1.3)	6.1	1.3 (1.0–1.7)	51.6	1.1 (1.0–1.3)
LADA	127	56.7	1.1 (0.8–1.6)	5.5	1.2 (0.5–2.6)	51.2	1.2 (0.8–1.7)
Unclassified	571	57.8	1.2 (1.0–1.5)	6.3	1.6 (1.1–2.3)	51.5	1.2 (1.0–1.4)
No, but glucose ≥ 11.1	216	45.4	0.7 (0.5–0.9)	5.1	0.8 (0.4–1.5)	40.3	0.7 (0.5–0.9)
							
**Serum glucose**							
≤ 4.7 mmol/l	16,198	43.2	1.0 (reference)	4.8	1.0 (reference)	38.3	1.0 (reference)
4.8–5.2 mmol/l	19,151	46.7	1.0 (0.9–1.0)	5.2	1.0 (0.9–1.1)	41.4	1.0 (0.9–1.0)
5.3–5.8 mmol/l	14,737	47.9	0.9 (0.9–1.0)	5.1	0.9 (0.8–1.0)	42.8	0.9 (0.9–1.0)
≥ 5.9 mmol/l	14,316	49.1	0.9 (0.8–0.9)	5.0	0.8 (0.7–0.9)	44.2	0.9 (0.8–0.9)
Missing	383	36.6	0.6 (0.5–0.8)	0.8	0.1 (0.0–0.4)	35.8	0.6 (0.5–0.8)
P-trend value^$^			< 0.001		< 0.001		< 0.001
P trend value^$ ^(β)			< 0.001 (-,022)		0.045 (-,026)		< 0.001 (-,021)

# Adjusted for age, gender, body mass index, and level of physical activity

$ Serum glucose treated as a single ordinal variable (categories 1 to 4 based on quartiles)

§ Serum glucose treated as a continuous variable (β value given in brackets)

Among the group of 216 individuals with non-fasting glucose ≥ 11.1 (but not known DM), chronic MSCs was less likely (OR = 0.7, 95% CI 0.5–0.9) than among those with no DM (Table 2). Furthermore, there was a linear trend (p < 0.001) of decreasing prevalence of chronic MSCs with increasing non-fasting glucose categories, also evident when glucose was treated as a continuous variable (Table 2). The prevalence OR of chronic widespread MSCs was 0.8 (95% CI 0.7–0.9) for individuals with glucose ≥ 5.9 mmol/l compared to those with glucose ≤ 4.7 mmol/l (Table 2). Thus, when adjusting for non-fasting serum glucose, chronic widespread MSCs was 1.6 times more likely (OR = 1.6, 95% CI 1.2–2.2) among subjects < 60 years of age with DM than among those without.

Chronic widespread MSCs were more likely among individuals with type 2 DM (OR = 1.3, 95% CI 1.0–1.7) and unclassified DM (OR = 1.6, 95% CI 1.1–2.3) than among those without DM (Table 2). When adjusting for non-fasting glucose, the corresponding ORs increased to 1.4 (95% CI 1.1–1.8) and 1.7 (95% CI 1.2–2.5) respectively. Among women, the strongest association was found with unclassified DM (OR = 2.1, 95% CI 1.3–3.3), whereas for type 2 DM among men (OR = 1.5, 95% CI 1.0–2.2). When merging individuals with type 2 DM and unclassified DM, chronic widespread MSCs were 1.5 times more likely (OR = 1.5, 95% CI 1.2–1.9) for this combined group compared to those without DM. The corresponding OR for those < 60 years was 1.8 (1.3–2.5). No significant association was found between chronic MSCs and type 1 DM or latent autoimmune diabetes of the adult (LADA) (Table 2).

Among the group of 1940 individuals with DM, no clear association was found between chronic MSCs and neither non-fasting serum glucose, HbA_1c_, nor duration of DM when treated as a single ordinal variable (Table 3). However, when HbA_1c _was treated as a continuous variable, there was a linear trend (p < 0.036, β = -,055) of decreasing prevalence of chronic non-widespread MSCs with increasing HbA_1c_.

**Table 3:** Prevalence OR^# ^of musculoskeletal complaints (MSCs) related to fasting serum glucose, HbA1c and duration of the disease in patients with DM

Variables	Total	Chronic MSCs		Chronic widespread MSCs		Chronic non-widespread MSCs	
	1940	%	OR (CI)	%	OR (CI)	%	OR (CI)
**Serum glucose**							
≤6.3 mmol/l	482	59.3	1.0 (reference)	6.4	1.0 (reference)	52.9	1.0 (reference)
6.4–8.7 mmol/l	487	55.2	0.8 (0.6–1.1)	5.1	0.7 (0.4–1.2)	50.1	0.8 (0.6–1.1)
8.8–12.1 mmol/l	472	54.9	0.8 (0.6–1.1)	6.1	0.8 (0.5–1.4)	48.7	0.8 (0.6–1.1)
≥12.2 mmol/l	469	57.4	1.0 (0.7–1.3)	6.4	1.0 (0.6–1.8)	51.0	1.0 (0.7–1.3)
Missing	30	70.0	1.2 (0.5–3.0)	0.0	-	70.0	1.5 (0.6–3.6)
							
**HbA1c**							
≤6.8 mmol/l	509	57.2	1.0 (reference)	6.7	1.0 (reference)	50.5	1.0 (reference)
6.9–7.8 mmol/l	422	59.2	1.1 (0.9–1.5)	5.0	0.8 (0.4–1.5)	54.3	1.2 (0.9–1.5)
7.9–9.2 mmol/l	481	55.7	1.0 (0.7–1.2)	5.2	0.8 (0.5–1.4)	50.5	1.0 (0.8–1.3)
≥9.3 mmol/l	426	53.8	0.9 (0.7–1.2)	7.0	1.0 (0.6–1.7)	46.7	0.9 (0.7–1.2)
Missing	102	64.7	1.4 (0.9–2.1)	4.9	0.8 (0.3–2.4)	59.8	1.4 (0.9–2.3)
							
**Duration of DM**
≤2 years	502	56.2	1.0 (reference)	6.4	1.0 (reference)	49.8	1.0 (reference)
3–6 years	360	56.4	1.0 (0.8–1.4)	4.7	0.8 (0.4–1.4)	51.7	1.1 (0.8–1.4)
7–13 years	403	57.1	1.1 (0.8–1.4)	6.0	1.1 (0.6–2.1)	51.1	1.1 (0.8–1.5)
≥14 years	406	59.1	1.2 (0.9–1.6)	6.9	1.6 (0.9–2.8)	52.2	1.2 (0.9–1.6)
Missing	269	55.4	1.0 (0.7–1.3)	5.2	1.0 (0.5–2.1)	50.2	1.0 (0.7–1.3)

# Adjusted for age, gender, body mass index, and level of physical activity

$ Serum glucose treated as a single ordinal variable (categories 1 to 4 based on quartiles)

§Serum glucose treated as a continuous variable (β value given in brackets)

## Discussion and conclusion

In this large-scale population-based cross-sectional study a high non-fasting glucose was associated with a lower prevalence of chronic MSCs, whereas individuals with DM were more likely to report chronic widespread MSCs than those without DM.

The strength of this study was the large and unselected population. To the best of our knowledge, this is the first large-scale population-based study evaluating the influence of chronic MSCs on individuals with DM whose classification is based on results of blood samples with anti-GAD and C-peptide in addition to questionnaire-based data. Although the participation rate of 70% was high, one may question to what degree the results can be generalized. Because questions about musculoskeletal pain were only a few out of more than 200 health-related questions, there is probably no selection bias regarding chronic MSCs. However, the possibility of a recruitment bias regarding DM should not be ignored. Non-responders were more frequent among young persons and among very old [10]. Particular selection by health status was more likely among the elderly [14]. The non-response problems related to the HUNT study have been thoroughly discussed elsewhere [14].

The participants were not asked to distinguish between pain in the left and right side of the body, and therefore we could not use the American College of Rheumatology (ACR) definition of chronic widespread pain. The group of individuals with chronic widespread MSCs was probably heterogeneous, but it may be of some relevance that a self-reported diagnosis of fibromyalgia was more than 5 times more common among individuals with chronic widespread MSCs than in those without. In the present study DM was associated with an increased prevalence of chronic widespread MSCs. A high prevalence of fibromyalgia has been reported among 100 patients with DM, both in type 1 and type 2, and a positive correlation between higher levels of HbA1c and more tender points [15]. Similarly, fibromyalgia was more likely among women with type 2 DM compared to controls [16], and the majority of a group of Italian patients with DM reported chronic musculoskeletal pain [17]. In the present study we found the strongest association between chronic widespread MSCs and the combined group of individuals with type 2 DM or unclassified DM. The positive relationship between type 2 DM and chronic widespread MSCs found in the present study raises the question whether type 2 DM may in some ways worsen chronic widespread MSCs, or vice versa. The causality issue cannot be properly addressed in a cross-sectional study. Age, overweight, and physical inactivity are strong risk factors for type DM [18], but we adjusted for all these factors in the analysis, and potential confounding was also evaluated for other life-style factors such as smoking and alcohol use. However, one can not rule out the possibility that there may be other unmeasured life-style factors or other factors incompletely registered that could influence our findings. For example, among those with known DM, anxiety and other psychological factors may influence their response to the questions about MSCs. One may also speculate that attempts to keep the glucose level low may influence the occurrence of chronic MSCs. DM affects vascular reactivity [19,20], and induces diabetic neuropathy [21], but because no association was found between chronic MSCs and type 1 DM or LADA, other causes than vascular changes may probably explain our main findings.

To the best of our knowledge, this is the first study evaluating the influence of non-fasting glucose on chronic MSCs in the general population. A subgroup of 216 individuals had non-fasting glucose ≥ 11.1, but they were not aware of DM at the time of the blood sampling. Therefore, anxiety for DM probably did not influence their response to the questions about MSCs. We found that hyperglycemia was associated with lower prevalence of chronic MSCs. Thus, our findings did not indicate that hyperglycemia *per se *increased the risk of chronic pain, at least in a short time perspective. Previously, several studies have reported reduced pain thresholds in hyperglycemia, but these studies have mainly been restricted to patients with known DM or diabetic animals [9,22-24]. In the present study no consistent association was found between non-fasting glucose, HbA_1c_, and prevalence of chronic MSCs among those with known DM. Thus, our results did not indicate that poor control of DM increases the prevalence of chronic MSCs as compared to good control defined as low HbA_1c _or low non-fasting glucose level.

In the present study high non-fasting glucose was associated with a lower prevalence of chronic MSCs, which may provide clues to the pathophysiology of chronic MSCs. High glucose levels or poor control of DM can of course not be recommended in a public health perspective.

## Competing interests

The authors declare that they have no competing interests.

## Authors' contributions

OMH, and KH conceived of the study and performed the statistical analysis. OMH, KM, JAZ and KH all participated in the design and drafted the manuscript. KM planned and was responsible for collection of data on diabetes. All authors read and approved the final manuscript.

## Pre-publication history

The pre-publication history for this paper can be accessed here:


